# Preoperative embolization of a solitary bone plasmacytoma of the proximal humerus

**DOI:** 10.1016/j.radcr.2024.09.042

**Published:** 2024-09-18

**Authors:** Parker Penny, Trevor Lin, Joyce Zhu, Brenden Li, Jonathan Henning, Austin Chao, Eusha Hasan, Joe Khoury, Mustafa Al-Roubaie

**Affiliations:** a Morsani College of Medicine, University of South Florida, 560 Channelside Dr, Tampa, FL 33602, USA; b Donald and Barbara Zucker School of Medicine at Hofstra/Northwell, 500 Hofstra Blvd, Hempstead, NY 11549, USA; c Moffitt Cancer Center, University of South Florida Morsani College of Medicine, 12902 USF Magnolia Drive, Tampa, FL, 33612, USA

**Keywords:** Plasmacytoma, Embolization, Preoperative, Pathologic fracture

## Abstract

Solitary plasmacytoma of bone (SPB) is a rare plasma cell malignancy that most often presents with localized pain. This case describes a 70-year-old female with a pathologic humeral fracture due to a large, hypervascular SPB. The tumor was assumed to be a metastatic lesion, and preoperative embolization was performed to minimize intraoperative blood loss, followed by tumor debulking and total shoulder arthroplasty. The total estimated blood loss was limited to 100cc, and the patient was returned to baseline functional status with full shoulder range of motion at 6 months postop. Literature on embolization of appendicular plasmacytomas is sparse; however, this case supports its efficacy. We recommend considering preoperative embolization as an adjunctive therapy for all hypervascular bone tumors requiring surgical management, regardless of origin.

## Introduction

Solitary plasmacytoma of bone (SPB) is a rare plasma cell malignancy without evidence of systemic disease. The incidence in the United States is less than 450 cases annually [1]. On a global level, the prevalence of this cancer is unclear. However it is thought these neoplasms account for less than 5% of patients with plasma cell myeloma [2]. The most common presenting symptom is localized pain, with pathologic fracture accounting for only 6% of presentations. Total 75% of SPBs occur within the axial skeleton while only 7% are found in long bones [3]. The characteristic radiographic appearance of SPB is a nonspecific solitary expansile lytic lesion with thinning and destruction of the cortex [4,5].

The standard treatment for all solitary plasmacytomas is radiation therapy with adjuvant chemotherapy reserved for patients with persistent disease. Extramedullary plasmacytomas can be resected if large and with well-defined borders. However, SPB resection should be reserved for patients with secondary complications like pathological fractures, neurological issues, or large lesions at high risk for these complications [6]. SPBs may be hypervascular, posing a challenge for surgical fixation of long bones.

The objective of this case report is to describe the successful application of preoperative embolization as an adjunctive technique to minimize intraoperative blood loss in the surgical management of a solitary plasmacytoma of bone and to suggest that the same technique should be considered in all hypervascular tumors, regardless of origin.

## Case

**Fig. 1:**
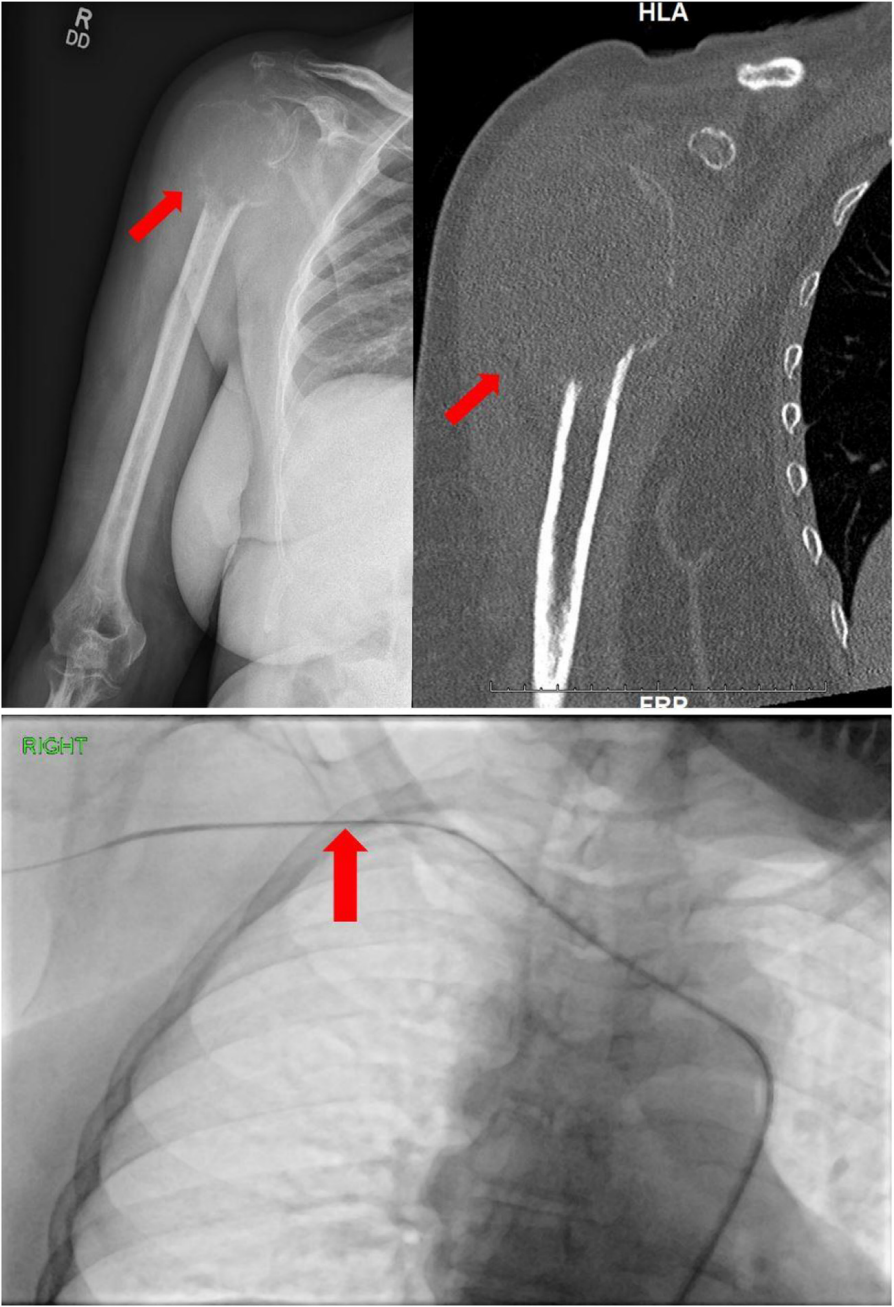
(A) Frontal x-ray and (B) coronal CT of the right shoulder show a large expansile lytic tumor in the humeral head, with an associated pathologic axial fracture of the surgical neck (arrow). (C) Intraprocedural spot fluoroscopic image demonstrates a 5F angled catheter in the right axillary artery from a femoral approach (arrow).

**Fig. 2:**
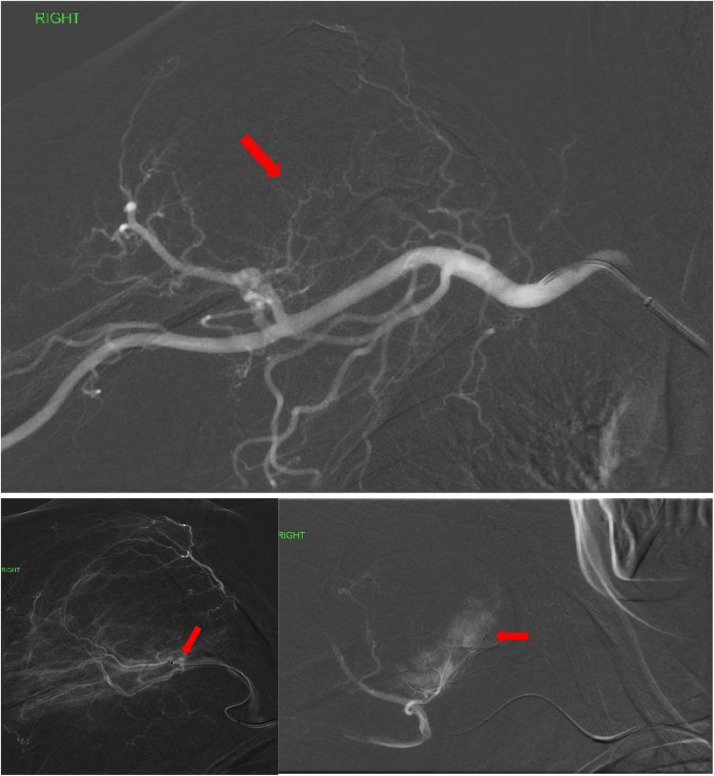
(A) Postprocessing digital subtraction angiography (DSA) of the right subclavian artery in the negative view demonstrates a hypervascular tumor involving the humeral head, with feeding arteries from multiple axillary and brachial artery branches (arrow). (B) DSA of a right humeral circumflex branch reveals robust arteriovenous fistulation within the tumor (arrow). (C) DSA of another humeral circumflex branch highlights the classic “tumor blush” appearance of malignancies (arrow).

**Fig. 3:**
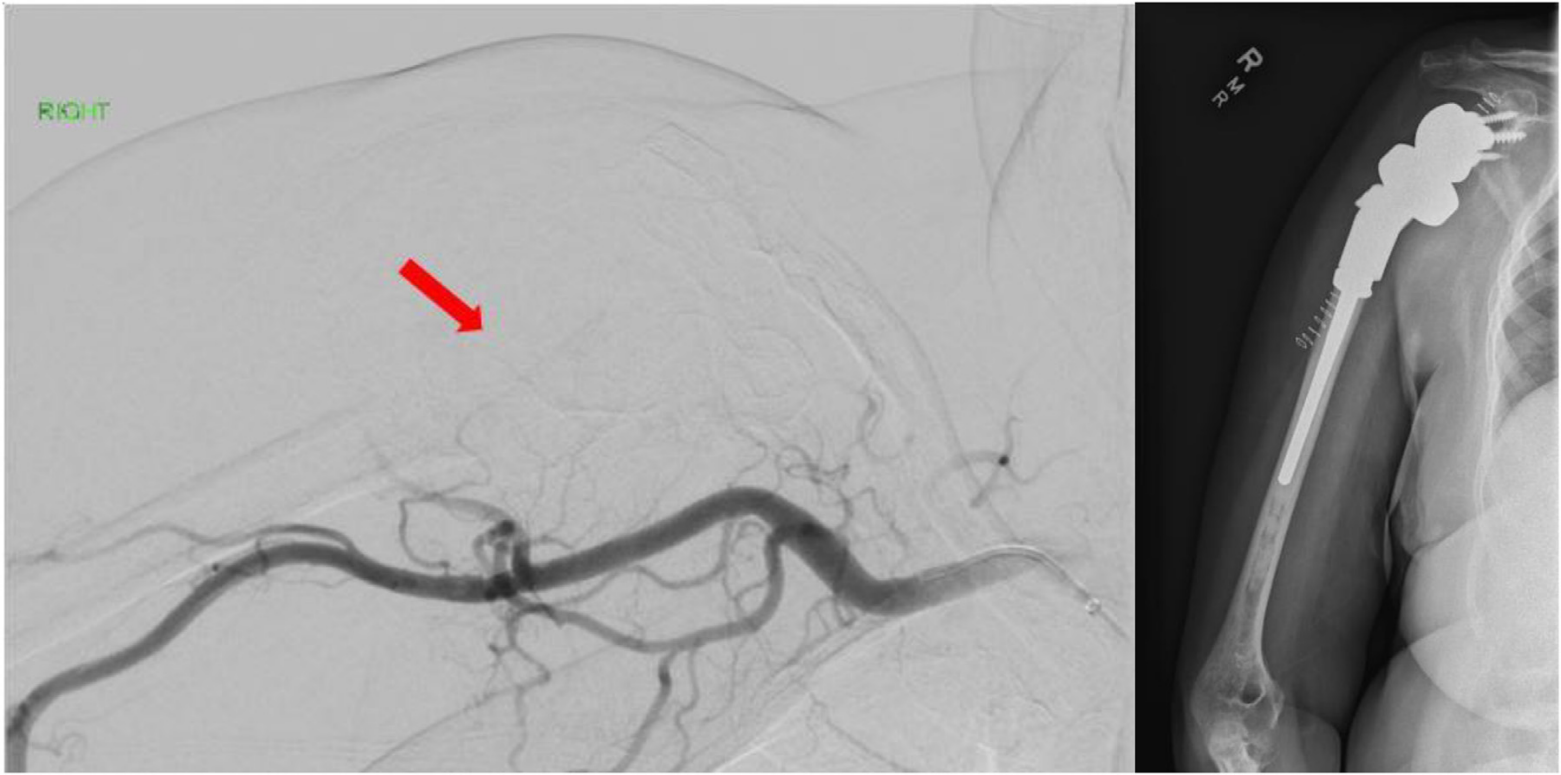
(A) Postembolization right subclavian artery DSA demonstrates cessation of flow to the tumor. (B) X-ray image of right total shoulder arthroplasty using a long intramedullary rod component.

**Fig. 4:**
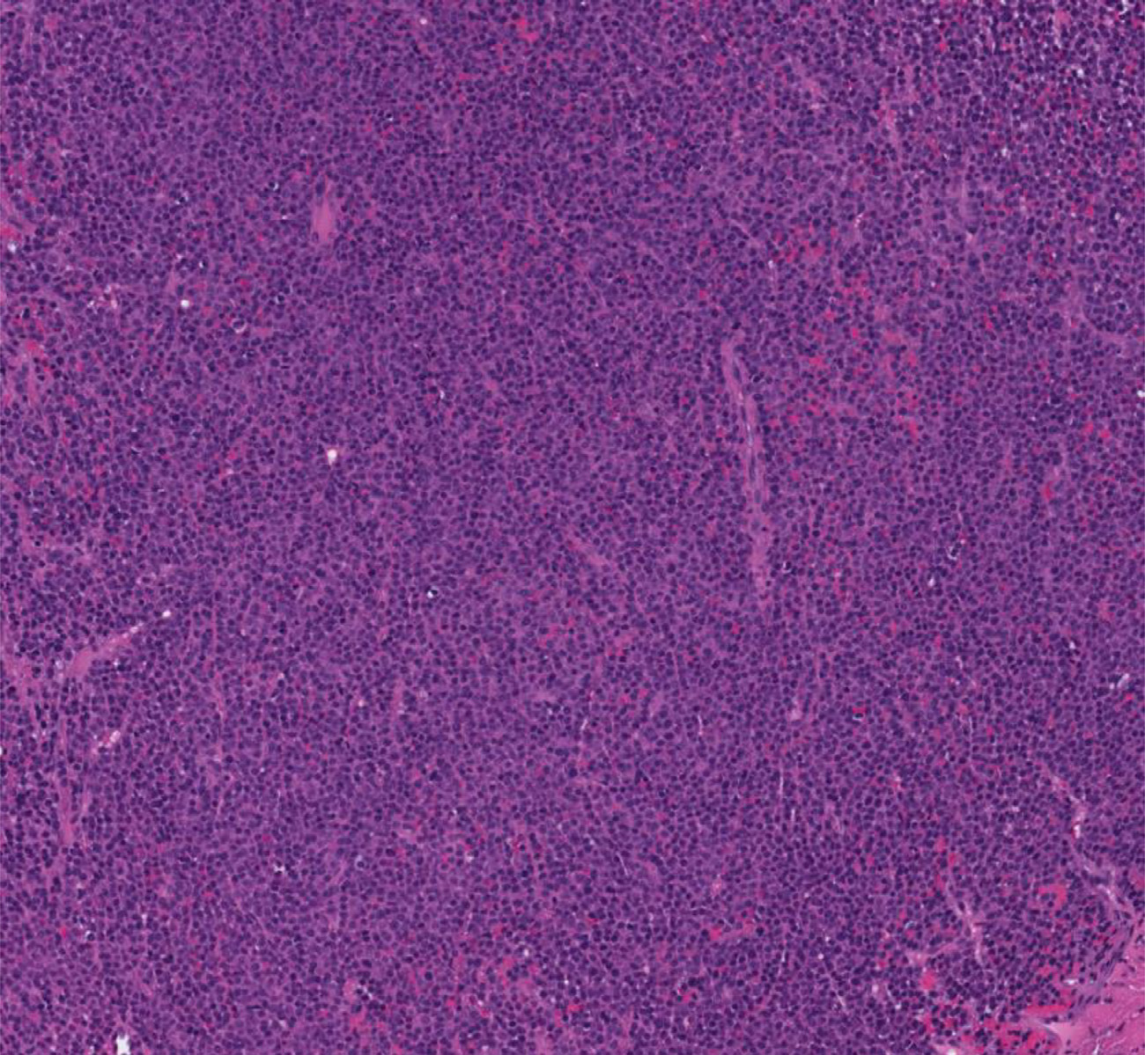
Surgical specimen from the resected tumor demonstrates immature monoclonal plasma cells with anaplastic morphology, high nuclear-to-cytoplasmic ratio, prominent nucleoli, and dispersed chromatin, consistent with plasmacytoma.

A 70-year-old African American female with hypertension, obesity and type 2 diabetes presented to the emergency department (ED) after a mechanical fall with severe right upper extremity pain. On exam, her right shoulder was severely tender to palpation, and range of motion was severely limited secondary to pain. An x-ray of her right shoulder demonstrated an acute fracture of the right humeral head associated with a large expansile lytic lesion (Fig. 1A), consistent with a pathologic fracture. A noncontrast CT of the right humerus was also performed, further characterizing the lytic and expansile nature of the humeral head tumor, measuring 4.3cm in greatest diameter (Fig. 1B). Given the urgent need for surgical fixation, the patient was referred to interventional radiology for preoperative embolization to minimize intraoperative blood loss. After a thorough discussion of the procedure and its risks, the patient was brought to the angiography suite, placed in the supine position and the right groin was prepped and raped in sterile fashion. Conscious sedation with continuous physiologic monitoring was provided by a IR nurse under the stewardship of the interventional radiologist. The right common femoral artery was accessed under ultrasound guidance. Fluoroscopic guidance was used for the remainder of the procedure. After placement of a 6F 90cm sheath into the thoracic aorta, a 5F angled catheter and glidewire were navigated into the aortic arch, then the brachiocephalic and right subclavian arteries, and ultimately positioned in the right axillary artery (Fig. 2). The 6F sheath was advanced over the wire and 5F angled catheter, and positioned securely within the right subclavian artery. Subsequently, a 2.4F microcatheter system was used to subselect multiple branches of the right humeral circumflex arteries supplying the hypervascular tumor. Angiography revealed robust arterial hypertrophy, tumor blushing as well as arteriovenous fistulation characteristic of malignant neovascularity (Fig. 2). No pseudoaneurysms were present. The tumoral vessels were each embolized using 100-300 micron particles in order to achieve distal embolization deep within the capillary bed of the tumor, and therefore minimize revascularization of the tumor from collateral vessels (Fig. 3). Particles were infused into the tumor methodically, and frequent digital subtraction angiograms were performed to avoid nontarget embolization into the normal tissues of the extremity. The presence of robust tumoral arteriovenous fistulization (AV) precluded the use of liquid embolic agents such as n-BCA glue due to the risk of nontarget embolization into the central venous system. After adequate devascularization of the tumor was confirmed on angiography, the catheters and sheaths were removed and hemostasis was achieved at the right common femoral artery access site with manual pressure. The patient was subsequently transferred to the operating room for tumor debulking and total right shoulder arthroplasty with a long intramedullary rod component (Fig. 3). The estimated blood loss during surgery was 100 cc. The patient had an uneventful 2-day postoperative recovery in the hospital and was subsequently discharged home. Histopathological examination of the resected specimen revealed a poorly differentiated plasma cell tumor (Fig. 4). The patient was referred to oncology for serological and imaging work-up to guide future management. After a 2-week recovery, the patient began a 2-month course of physical therapy. At 6 months postop, the patient reports normal baseline functional status and restored right shoulder range of motion.

## Discussion

The current level of evidence supporting preoperative embolization of SPBs is low and primary based on case reports and small comparative studies of nonspecific hypervascular tumors. This case demonstrates the role for preoperative embolization of SPBs. The goal of embolization is to selectively devascularize the tumor while preserving physiologic arteries. Reducing tumor vascularity assists surgeons in safely obtaining adequate excision margins and reducing intraoperative blood loss by both reducing the need for intraoperative hemostasis and by causing tumor ischemia, which may reduce the size of the tumor [7].

This patient presented to the ED with an acute pathologic fracture of the humerus, which is 500 times more likely to be from metastatic malignancy in patients over 40 than from a primary bone lesion [8]. Typically, renal cell carcinoma (RCC) metastasis is the most common hypervascular metastatic bone tumor seen in clinical practice [9]. Preoperative embolization of these tumors is a controversial topic. While some studies demonstrated intraoperative blood loss reductions between 290 mL and 1000 mL compared to the no-embolization groups, others showed no difference in blood loss or transfusion requirements between the 2 groups. The level of clinical evidence supporting this intervention for metastatic RCC is low. However, these studies are all retrospective cohorts with no standardization of tumor location or type of surgery, nor any comparison to a matched untreated group [10,11]. Therefore, the decision to utilize preoperative embolization for these metastatic tumors is often center-dependent, and patient specific.

While there have been documented cases of solitary humeral head plasmacytomas [12,13], there is sparse literature regarding perioperative management of these appendicular tumors, likely due to the rarity of presentation. However, embolization of hypervascular humeral lesions similar to those of our patient is well documented. The adjuvant therapy has been shown to be useful in humeral giant cell tumors, bone cysts, and metastasis [[14], [15], [16], [17], [18]]. It has been suggested that embolization techniques typically used for metastatic lesions can similarly be used in primary tumors [19,20]. One study compared the embolization outcomes of hypervascular RCC metastatic lesions to primary bone lesions and found no difference in perioperative blood loss [21].

The literature on embolization of plasmacytomas is mostly limited to the axial skeleton. Two studies compared preoperative embolization with intraoperative local hemostatic agent use for hypervascular spinal tumors. Both found that embolization is useful for reducing blood loss for spondylectomy of these types of tumors, including plasmacytomas. However, there was no advantage when performing palliative decompression [9,22]. One case report showed a favorable outcome and reduced blood loss for embolization before fixation of a spinal plasmacytoma [23]. Two other case reports showed the successful use of embolization before resection of cranial plasmacytomas [24,25].

While in some cases there appears to be a surgical benefit to successful preoperative embolization of both primary and metastatic bone lesions, the procedure is not without risk. Transient nerve palsies, soft tissue abscesses, vascular injury, and postembolization syndrome are some of the possible complications that have been described [10,[26], [27], [28], [29]]. Using computed tomography angiography for pre-embolization mapping and selecting appropriately sized particles to avoid compromising arterial supply below the level of collaterals are commonly used strategies to reduce the risk of the procedure [30]. In this case, extensive mapping of the tumor arterials was accomplished preoperatively. The extensive AV fistulization of the tumor made solid particles the preferred choice over liquid agents. Digital subtraction angiograms were performed throughout the case to minimize the risk of embolizing physiologic tissues of the arm. However, each lesion must be evaluated on a case-by-case basis to determine the relative risk of embolization, particularly for those in the extremities.

## Conclusion

Our experience with the preoperative embolization of a humeral solitary plasmacytoma supports the broader application of this adjunctive technique for hypervascular tumors. The positive outcomes observed in this case, including extensive reduction in malignant vascularity and minimal intraoperative blood loss, indicate that treating hypervascular tumors similarly, regardless of their origin, can be efficacious. Therefore, we recommend that embolization should be a standard consideration for all hypervascular tumors of bone, both primary and metastases, similar to the approach often taken with RCC metastasis. Future research should establish guidelines for the use of preoperative embolization in the management of hypervascular bone tumors using prospective randomized studies.

## Patient consent

Informed written consent was obtained from the patient with regards to publishing their medical course for the purposes of medical education.

## References

[1] Thumallapally N. (2017). Solitary plasmacytoma: population-based analysis of survival trends and effect of various treatment modalities in the USA. BMC Cancer.

[2] Dimopoulos M.A., Hamilos G. (2002). Solitary bone plasmacytoma and extramedullary plasmacytoma. Curr Treat Options Oncol.

[3] Agbuduwe C. (2020). Clinical presentation and outcomes of solitary plasmacytoma in a tertiary hospital in the UK. Clin Med (Lond).

[4] Grammatico S., Scalzulli E., Petrucci M.T. (2017). Solitary Plasmacytoma. Mediterr J Hematol Infect Dis.

[5] Di Muzio B., A.A., Ibrahim D, et al. Solitary bone plasmacytoma. Reference article [cited 2024 Jul 24]; Available from: https://radiopaedia.org/articles/solitary-bone-plasmacytoma-1?lang=us.

[6] Caers J. (2018). Diagnosis, treatment, and response assessment in solitary plasmacytoma: updated recommendations from a European Expert Panel. J Hematol Oncol.

[7] Lee V.N. (2008). Preoperative embolisation in benign bone tumour excision. J Orthop Surg (Hong Kong).

[8] Biermann J.S. (2009). Metastatic bone disease: diagnosis, evaluation, and treatment. J Bone Joint Surg Am.

[9] Ptashnikov D. (2014). Preoperative embolization versus local hemostatic agents in surgery of hypervascular spinal tumors. Int J Spine Surg.

[10] Geraets S.E.W., Bos P.K., van der Stok J. (2020). Preoperative embolization in surgical treatment of long bone metastasis: a systematic literature review. EFORT Open Rev.

[11] Amado A.A. (2023). Embolization of renal cell carcinoma skeletal metastases preceding orthopedic surgery. Cureus.

[12] Tandon S. (2022). Solitary bone plasmacytoma of humerus presenting as a nonhealing fracture in a child: a rare entity. J Pediatr Hematol Oncol.

[13] Ly J.Q., Sandiego J.W., Beall D.P. (2005). Plasmacytoma of the proximal humerus. Clin Imaging.

[14] Emori M. (2012). Pre-operative selective arterial embolization as a neoadjuvant therapy for proximal humerus giant cell tumor of bone: radiological and histological evaluation. Jpn J Clin Oncol.

[15] Mascard E., Gomez-Brouchet A., Lambot K. (2015). Bone cysts: unicameral and aneurysmal bone cyst. Orthop Traumatol Surg Res.

[16] Cardinale U. (2023). Inverse shoulder tumor megaprosthesis after large bone resection in massive metastasis of the proximal humerus. Acta Biomed.

[17] Layalle I. (1998). Arterial embolization of bone metastases: is it worthwhile?. J Belge Radiol.

[18] Pan P. (2019). Ipsilateral transradial access in transarterial embolization of upper extremity bony metastases. J Vasc Access.

[19] Macedo F. (2017). Bone metastases: an overview. Oncol Rev.

[20] Haber Z. (2023). Transarterial embolization of bone metastases. Tech Vasc Interv Radiol.

[21] Sare A. (2021). Perioperative blood loss after embolization of hypervascular musculoskeletal tumors outside of the spine: a single-center ten year experience and systematic review of the literature. Clin Imaging.

[22] Zaborovsky N.S. (2016). [Prevention of blood loss during resection of hypervascular spinal tumors with the use of preoperative embolization and local hemostatic agents]. Vopr Onkol.

[23] Binh N.T. (2022). Preoperative embolization of hypervascular spinal tumors: two case reports. J Clin Imaging Sci.

[24] Harada K. (1991). [Plasma cell tumor of the parieto-occipital bone; a case report]. No Shinkei Geka.

[25] Nakano H. (2017). [A case of a solitary plasmacytoma extending into the extradural space]. No Shinkei Geka.

[26] Kickuth R. (2008). Interventional management of hypervascular osseous metastasis: role of embolotherapy before orthopedic tumor resection and bone stabilization. AJR Am J Roentgenol.

[27] Finstein J.L. (2006). Postembolization paralysis in a man with a thoracolumbar giant cell tumor. Clin Orthop Relat Res.

[28] Kim W. (2015). Preoperative embolization for bone metastasis from hepatocellular carcinoma. Orthopedics.

[29] Sun S., Lang E.V. (1998). Bone metastases from renal cell carcinoma: preoperative embolization. J Vasc Interv Radiol.

[30] Lau V., Sun M., Chu F. (2013). Embolisation of hypervascular bone tumours: a pictorial essay with literature review. J Med Imaging Radiat Oncol.

